# Anévrysme de la veine de Galien révélé à l’âge adulte

**DOI:** 10.11604/pamj.2014.19.102.5212

**Published:** 2014-09-29

**Authors:** Badreeddine Alami, Mustapha Maaroufi

**Affiliations:** 1 Service de Radiologie, CHU Hassan II, Fès, Maroc

**Keywords:** Anévrysme de la veine de Galien, affection congénitale, malformations artério-veineuses intracrâniennes, Aneurysm of the vein of Galen, congenital disorder, intracranial arteriovenous malformations

## Image en medicine

L'anévrysme de la veine de Galien est une affection congénitale rare, représentant moins de 1 % des malformations artério-veineuses intracrâniennes. Il résulte de l'absence de différenciation en système artério-veineux mature, entre 7 et 12 SA, d'une veine embryonnaire primitive, médiane qui draine les plexus choroïdes primitifs. La majorité des cas de malformation anévrysmale de la veine de Galien sont diagnostiqués en périodes anténatale, néonatale ou post- natale, le diagnostic à l'âge adulte est exceptionnel. Le tableau cliniqueà tout âge peut inclure: insuffisance cardiaque, retard psychomoteur, hydrocéphalie et crises d'épilepsie. Le traitement de référence est l'embolisation réalisée à quelques mois de vie mais le pronostic reste redoutable. Nous rapportons le cas d'un homme, âgé de 38 ans, admis aux urgences dans un tableau d'état de mal épileptique avec un syndrome méningé à l'examen clinique. Une TDM cérébrale a objectivé unemasse de la région pinéale, présentant des calcifications périphériques, rehaussé intensément après injection du produit de contraste en rapport avec une volumineuse dilatation anévrysmale de la grande veine de Galien. L'IRM cérébrale a montré cet anévrysme qui mesure 50 x 50 x 40 mm, associé à une dilatation du sinus droit et de multiples dérivations veineuses péri-anévrysmales avec épaississement et rehaussement dure-mérien régulier et diffus en rapport avec une méningite bactérienne confirmée par le bilan biologique. Vu qu'il n'y avait pas de signes d'hypertension intracrânienne ni d'hydrocéphalie, le patient a reçu un traitement médical de sa méningite associé à un traitement antiépileptique avec une bonne évolution clinique.

**Figure 1:**
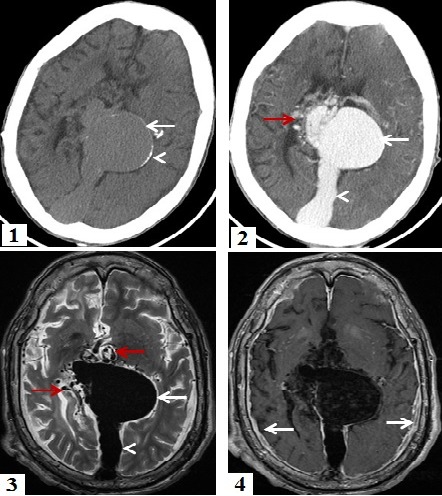
TDM cérébrale en Coupe axiale, sans injection du produit de contraste montrant une masse de la région pinéale (flèche) avec calcification périphérique (tête de flèche). (2) : TDM cérébrale en Coupe axiale, après injection du produit de contraste iodé objectivant un rehaussement intense de type vasculaire de la masse (flèche blanche) associé à une dilatation du sinus droit (tête flèche) et des dérivations vasculaires périlésionnelles (flèche rouge). (3) : IRM cérébrale, coupe axiale en séquence T2 montrant un anévrysme de la veine de Galien (flèche blanche) associé à la dilatation du sinus droit (tête flèche) et des dérivations péri-anévrysmales (flèche rouge). (4) : IRM cérébrale, coupe axiale en séquence T1 après injection du gadolinium objectivant en plus de l'anévrysme de la veine de Galien un épaississement avec rehaussement dure-mérien régulier et diffus (flèche)

